# Thrombopoietin mimetic therapy alleviates radiation-induced bone marrow vascular injury in a bone marrow transplant mouse model

**DOI:** 10.3389/fonc.2024.1414488

**Published:** 2024-10-10

**Authors:** Hemendra Ghimire, Srideshikan Sargur Madabushi, Justin Vercellino, Jamison Brooks, Darren Zuro, Ji Eun Lim, Paresh Vishwasrao, Amr Mohamed Hamed Abdelhamid, Guy Strome, Gary Eichenbaum, Monzr Al Malki, Chandan Guha, Susanta K. Hui

**Affiliations:** ^1^ Department of Radiation Oncology, City of Hope National Medical Center, Duarte, CA, United States; ^2^ Department of Radiation Oncology, Montefiore Medical Center, Bronx, NY, United States; ^3^ Department of Pathology, Albert Einstein College of Medicine, Bronx, NY, United States; ^4^ Department of Radiation Oncology, Mayo Clinic, Rochester, MN, United States; ^5^ Department of Radiation Oncology, University of Oklahoma Health Sciences Center, Oklahoma City, OK, United States; ^6^ Department of Clinical Oncology and Nuclear Medicine, Faculty of Medicine, Ain Shams University, Cairo, Egypt; ^7^ Department of Radiotherapy, Universitair Ziekenhuis (UZ) Brussels, Brussels, Belgium; ^8^ Johnson and Johnson, Office of the Chief Medical Officer, New Brunswick, NJ, United States; ^9^ Department of Hematology and Hematopoietic Cell Transplantation, City of Hope National Medical Center, Duarte, CA, United States

**Keywords:** bone marrow transplantation, confocal microscopy, intravital multiphoton microscopy, thrombopoietin mimetic, x-ray irradiation

## Abstract

**Background:**

There is a need for therapies that can mitigate bone marrow dysfunction and organ toxicity that occur following myeloablative injury and reduced intensity conditioning regimens used in patients undergoing bone marrow transplantation (BMT). The pathogenesis of adverse effects from BMT conditioning has been linked to injury to the vascular endothelium, bone marrow (BM), and other organs.

**Objective:**

To evaluate the impact of the thrombopoietin mimetic drug JNJ-26366821 (TPOm) on BM vascular recovery in mice undergoing myeloablative radiation conditioning followed by BMT.

**Study design:**

TPOm (doses: 0 µg, 300 µg, 1000 µg per Kg body weight) was administered on Days 0 and 7 after BMT, in mice receiving a total body irradiation (TBI) conditioning regimen (5.5 Gy x 2) before congenic BMT. BM donner cell engraftment was analyzed using flow cytometry on Days 7, 14, and 30 post-BMT. The morphological and biophysical properties of the BM vasculature were evaluated by intravital multiphoton microscopy (MPM) and immunofluorescence confocal imaging. Herein, morphological properties involve microvascular density (MVD), vessel diameter, and vascular area, while biophysical properties include transfer rate (K_trans_) of contrast within the BM vascular niche, as well as the fractional volume (*v_ec_*) of extracellular extravascular tissue (EES).

**Results:**

No significant difference in donor chimerism was observed at days 7, 14, and 30 post-BMT, between TPOm and PBS-treated mice. TPOm intervention improved BM vasculature regeneration in transplanted mice. The MVD, K_trans,_ and BM vasculature as well as vascular endothelial growth factor receptor-2 (VEGFR2) in the BM, showed a dose dependent improvement in mice treated with TPOm. On day 14 post-BMT, the group receiving 1000 µg/Kg TPOm showed significant shifts (p-value < 0.05) in MVD, K_trans_, and VEGFR2 expression from their corresponding control types (TPOm dose 0 µg) towards levels comparable to healthy controls.

**Conclusion:**

TPOm intervention augments BM vascular structure and function, which may be important for hematopoietic recovery and bone marrow function in radiation conditioned hematopoietic stem cell transplant patients, in addition to enhancing platelet recovery.

## Introduction

Conditioning regimens prior to hematopoietic stem cell (HSC) or bone marrow (BM) transplantation are used to prevent graft rejection and reduce disease burden (1). This preparative procedure often induces toxicity, which can have a significant impact on morbidity and mortality (2). Myeloablative conditioning regimens generally provide enhanced reduction in disease burden compared to reduced intensity conditioning regimens (2), but can cause long-lasting damage to the BM constituents and potential delays in immune reconstitution (3). The delayed regeneration of the BM vasculature and its microenvironment can limit the recovery of hematopoiesis (4) and provide an environment for new diseases to emerge. Therefore, rapid and effective restoration of the hematopoietic and vascular systems could provide significant therapeutic benefits (5).

Myeloablative conditioning prior to bone marrow transplantation (BMT) can be achieved with chemotherapy and/or radiotherapy using total body irradiation (TBI). Myeloablative radiation regimens besides eradicating diseased cells also eliminates host hematopoietic and immune cells thereby preventing graft rejection, which is essential for a successful treatment for patients with malignant [like acute myeloid leukemia (AML) or acute lymphoid leukemia (ALL)] and non-malignant hematological diseases (Sickle cell disease) undergoing BMT (6). Although increasing radiation dose could be beneficial in disease eradication but apparent radiation-induced organ toxicity in TBI limits further intensification of transplantation conditioning. Improved radiation modalities like total marrow irradiation (TMI) (7, 8), and total marrow and total lymphoid irradiation (TMLI) (9) are also being evaluated for their potential to control disease in the BM through dose-escalation while limiting dose to the vital organs like lung, liver, gut, etc. (10). The potential of TMI and TMLI techniques to improve treatment outcomes further warrants the evaluation of methods to facilitate BM repair after radiotherapy (11–13).

Several pharmacological agents with different targets and mechanisms are being studied to mitigate and treat the toxicity induced by ionizing radiation, and to expedite hematopoietic and vascular restoration after BMT (14). These include probiotics, immunomodulators, metabolism modulators, antioxidants, anti-inflammatory agents, DNA repair modulators, and hematopoietic growth factors (15). Thrombopoietin (TPO), an endogenous hematopoietic cytokine, is a glycoprotein hormone that is produced primarily in the liver and kidneys, which plays a crucial role in platelet production and therefore essential in platelet recovery to prevent thrombocytopenia post BMT. TPO stimulates the proliferation, differentiation, and maturation of megakaryocytes and regulates platelet production (16). Further, thrombopoietin and its receptor mimetics has been reviewed for its therapeutic efficacy of such potential drugs and their actions (16). Due to its potential to cause anti-platelet antibodies, recombinant human TPO therapy has not been used in clinical application (17). To overcome this issue, a number of TPO-mimetics, with no sequence homology to endogenous TPO, have been developed and approved to treat idiopathic thrombocytopenia purpura (ITP). Like endogenous TPO, these drugs increase platelet production by stimulating the receptor, c-Mpl. Further, TPO mimetics have been studied for their potential to mitigate hematopoietic syndrome of Acute Radiation Syndrome (ARS) due to their effects on megakaryocytes, platelets, and other HSC precursors (17, 18).

JNJ-26366821 (TPOm) is a novel pegylated peptide that, in addition to its platelet stimulating effects in preclinical (19) and clinical studies (20), has shown potential to mitigate radiation injury (21, 22), bone marrow injury (23), and overall survival of murine models through both hematopoietic and vascular protective/regenerative effects (24). Studies also indicate that JNJ-26366821 treatment increases megakaryopoiesis without affecting malignant myeloid proliferation in myelodysplastic syndrome (MDS) and acute myeloid leukemia (AML) (25). This selective effect is particularly valuable in BMT, where achieving a balance between hematopoietic recovery and controlling malignancy is crucial (26). Based on these data, we hypothesize that TPOm intervention will mitigate BM injury and promote vascular recovery following myeloablative conditioning post-BMT.

The intravital multiphoton microscopy (MPM), a non-invasive high resolution quantitative imaging biomarker tool, was shown to be useful measuring vascular and extravascular events in the frontal calvarium region were evaluated by using in real-time (27–29). Brooks et al. further used this technique to assess leukemia induced remodeling of bone marrow vasculature and ability of low dose radiation to enhance BM vascular permeability to support drug delivery, consequently improving survival (30, 31). In this study, we used MPM to evaluate whether TPOm mitigates injury and improves BM vascular regeneration in mice that underwent myeloablative radiation followed by BMT. We found the benefits of JNJ-26366821 treatment for enhancing both the morphological and physiological improvements in bone marrow microvasculature subsequent to myeloablative radiation conditioning for BMT. This data may provide rationale for clinical evaluation of the potential for TPOm to mitigate bone marrow toxicity in patients receiving chemotherapy and radiation conditioning.

## Materials and methods

All studies were performed in accordance with the guidelines and approval of the Institutional Animal Care and Use Committee at City of Hope National Medical Center. In this study, C57BL/6J mice (Strain 000664; Jackson Laboratory, Bar Harbor, ME) of 8-10 weeks male mice were used as host and B6.SJL-*Ptprc^a^ Pepc^b^*/BoyJ (Strain # 002014 B6 CD45.1, Jackson Laboratory, Bar Harbor, ME) as donor. The C57BL/6 mice were chosen for our study for their manageable size, higher radiation resistance, robust tail veins for contrast injections, and tolerance to anesthesia for longer experiments. Similarly, animal numbers were made based on ensuring sufficient sample sizes for meaningful data analysis while adhering to ethical guidelines for minimizing animal usage. A comparative analysis was performed between control/healthy mice and transplant mice with varying doses of TPOm intervention as outlined in **Table 1**.

**Table 1:** TPOm doses, TPOm treatment time, and BM vasculature evaluation days after BMT in studied mice groups.

Group		Treatment	TPOm Dose	Treatment Timing (Days after BMT)	Evaluation (Days after BMT)
Irradiation (day -1 and BMT (day 0)	PBS_D7	PBS	0 µg/Kg	0	7
	TPO300_D7	TPOm	300 µg/Kg	0	7
	PBS_D14	PBS	0 µg/Kg	0 and 7	16
	TPO300_D14	TPOm	300 µg/Kg	0 and 7	14
	TPO1000_D14	TPOm	1000 µg/Kg	0 and 7	14
Control		Healthy mice were imaged alongside the treated mice.			

Healthy mice that were not irradiated or treated with TPOm served as controls.

### X ray irradiation and radiation doses

X-RAD SmART – Precision X-ray irradiation, machine operated at 225 kVp, 13 mA, with 0.3 mm copper filter, was used. Unanesthetized mice were placed in individual chambers of a mouse pie restrainer for total body irradiation (TBI). Mice were irradiated with 11 Gy TBI (2-fractions, 6 hours apart, 5.5 Gy each), at the dose rate of 1.5 Gy/min on pie chamber placed at 58.57 cm from the source. In our previous study, we successfully demonstrated that 11 Gy is a myeloablative dose for TBI, enhancing donor cell engraftment in both primary and secondary BMT (32).

### Congenic BMT and TPOm administration

Stocks of JNJ-26366821 at concentrations of 60 µg/mL were prepared and stored at -80°C and diluted as needed. C57BL/6J host mice (CD45.2/H-2K^b^) were treated with TBI 11 Gy in 2 fractions of 5.5 Gy each 6 h apart. 24 h ± 1 h post-radiation and 30 mins - 1 h prior to BMT, were subcutaneously administered with TPOm 0 µg/kg, 300 µg/kg, or 1000 µg/kg. These TPOm doses were chosen based on previous studies with CD2F1 mice, which showed that doses of 300 µg/kg and 1000 µg/kg are well tolerated and safe for use in mouse models (19, 24).

BMT was performed on these mice by injecting 4 million whole BM cells isolated from CD45.1 donor mice (H-2K^b^) through an intravenous route via tail vein. The day 7 study group received only one dose of TPOm on the day of BMT, while the day 14 study group received an additional TPOm dose on day 7 after BMT. In all transplantation studies, the donors are CD45.1 and the recipients are CD45.2 C57BL/6J males.

### MPM imaging of the calvarium and image analysis

In this study, animal preparation for the MPM imaging and subsequent image analysis followed the established methods described in our previous publications (30, 31). The day before intravital imaging, a custom-built carbon head plate with an inner diameter of 8 mm was affixed directly to the frontal bone region of the calvarium using Pearson-PQ glass ionomer cement. Mice were anesthetized with 1-2% isoflurane at 0.5-1L/min O_2_ flow rate, during the imaging. A catheter with a 27-gauge needle with 0.025” Micro-Renathane^®^ tubing connected to an extension set was inserted into the mouse tail before loading mice onto the custom-built microscope stage maintained at 37°C for imaging procedure. This catheter facilitates tail vein infusions of contrast agents during the imaging.

A Prairie Ultima multiphoton microscope (Bruker Corporation, Billerica, MA), equipped with an Olympus XLUMPlanFL 20x objective (1.00 NA water objective) was used for image acquisition. Time-lapse imaging was performed with the intravenous injection of TRITC-dextran;150 kDa (TdB Consultancy, Uppsala, Sweden), at a dose of 350 µg/mouse, dissolved in 100 µL of PBS. Similarly, vascular blood pool imaging was achieved with the intravenous injection of 10 µL of Qtracker™ 655 Vascular Label (Invitrogen, Carlsbad, CA) added to 90 µL of PBS.

The average diameter and the number of vessel branches per area were performed with Fiji/ImageJ by analyzing images of Q-tracker, vascular label post-injection. Quantitative analysis of time-lapsed images of the contrast distribution reflects physiological parameters indicating the functional status of the vascular system or the dynamic nature of the tissue environment. Herein, contrast transfer rate (K_trans_) values, and the fractional volume (*v_ec_*) of extracellular extravascular tissue (EES), were determined by using Toft model, as previously described (30, 31).

### Whole-mount immunofluorescence imaging and analysis of femoral BM

Femoral bones were harvested and fixed overnight in 10% paraformaldehyde (PFA) at room temperature (to preserve the tissue components and morphology), followed by cryoprotection in 30% sucrose for at least 24 hours before embedding in O.C.T. compound for freezing. Femurs were cut transversely on a cryostat until the bone marrow cavity was fully exposed. Femurs were removed from the O.C.T compound by submerging embedded tissue in 1X PBS for 10 minutes. Bones were further washed in 1X PBS and transferred into Eppendorf tubes containing staining/blocking buffer (10% DMSO, 5% horse serum, 0.5% Triton X-100) overnight at 4°C (33). The following day, femurs were split horizontally into proximal and distal ends and moved to a new Eppendorf tube with primary antibodies in staining solution for 3 days at 4°C with goat anti-mouse VEGFR2 (R&D, AF644, 1:100) and mouse anti-mouse CD45.1 (Biolegend, 110750, 1:100). Femurs were washed in 1X PBS 3 times for 1 day, then stained with secondary antibodies in staining solution for an additional 3 days at 4°C with Alexa Fluor 488 AffiniPure F(ab’)_2_ Fragment Donkey Anti-Goat IgG (Jackson ImmunoResearch, 1:500). After the femurs were washed 3 times for 1 day and stained with Hoechst 33342 (Life Technologies, 1:2000) for 30 minutes at room temperature, followed by one more wash in PBS for 10 minutes.

Images were acquired using an upright ZEISS AXIO Examiner D1 microscope (Zeiss) with a confocal scanner unit, CSUX1CU (Yokogawa), and reconstructed in three dimensions with Bitplane Imaris v9.6.0 (33). Briefly, original images in Slidebook format were loaded into Imaris, and the detection of VEGFR2+ vessels was segmented with the “surfaces” module by voxel thresholding. The quantification of the vessel surface area was subsequently determined. Representative images were acquired through Velocity after normalization to naïve (control) and the noise reduction filter. The key resource table for the whole-mount immunofluorescence imaging and analysis is in **Supplementary Table S1**.

### Statistical analysis

Statistical analyses were performed using GraphPad Prism software (GraphPad Software Inc., La Jolla, CA, USA). Two-way ANOVA test (followed by Tukey’s multiple comparisons and Benjamini-Hochberg (BH) correction) were used, and the data are presented as mean ± SEM. Differences were considered significant when p-values were <0.05. Significance levels were indicated as follows: ns = not significant, *p < 0.05, **p < 0.01, ***p < 0.001, ****p < 0.0001. In addition, the discriminating potential is further tested by using sensitivity, specificity, and area under the curves (AUC) values under the receiver operating characteristic (ROC) curves.

## Results

### JNJ-26366821 administration improved platelet recovery and increased total donor BM stem and progenitor cells

**Figure 1:**
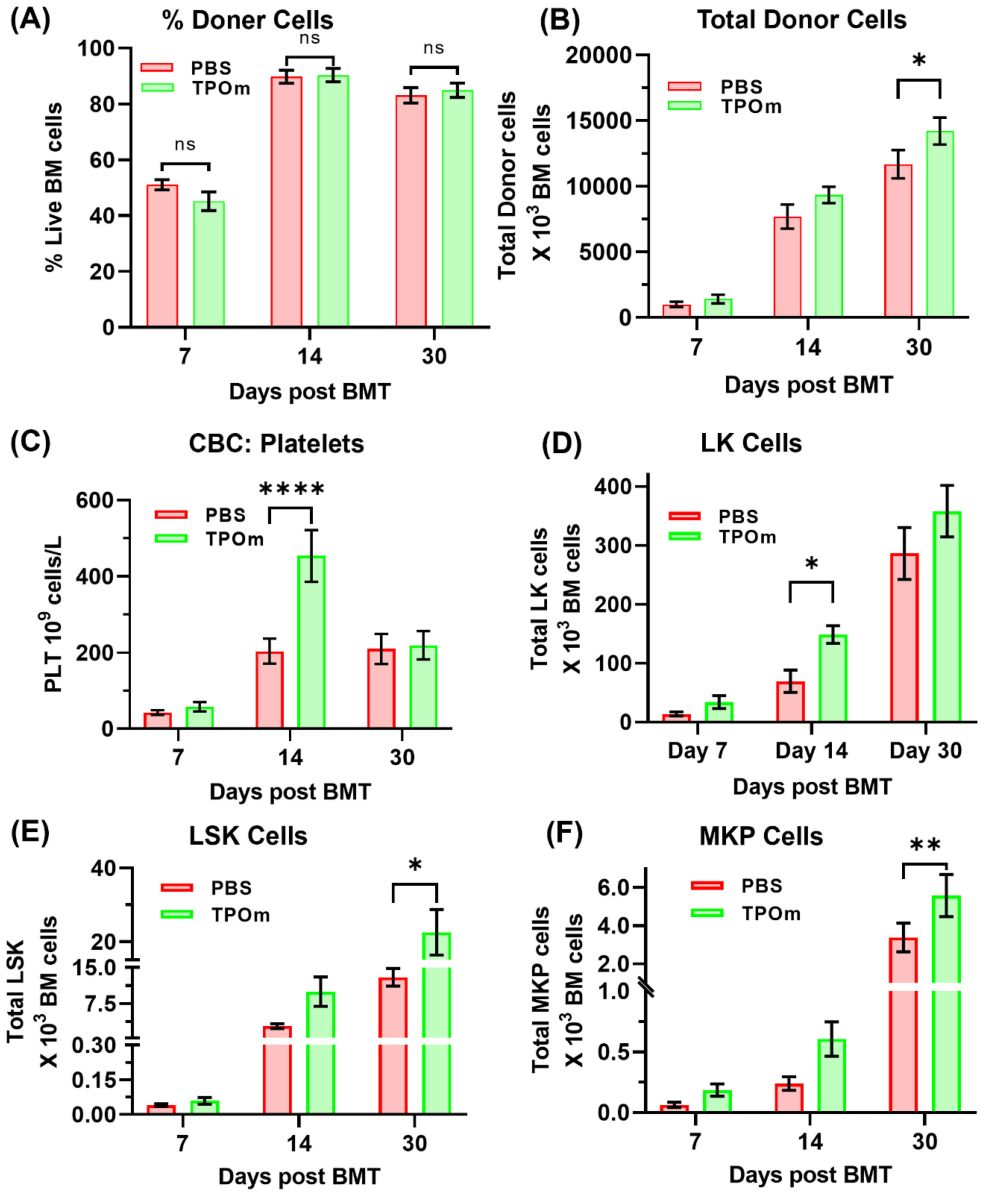
Effect of TPOm on hematopoietic recovery post-BMT. Mice were treated with PBS or TPOm on the day of radiation conditioning, and again on days 7 and 14 post-BMT. Hematopoietic recovery was assessed by peripheral blood CBC and donor chimerism by flow cytometry. **(A, B)** Percentage donor chimerism was similar between PBS and TPOm treated mice, with both groups showing ≥80% donor cells by day 14 post-BMT. However, total BM cellularity and total donor cells were significantly elevated in TPOm treated mice by day 30. **(C)** CBC analysis on Day 7, Day 14 and day 30 post BMT showed similar profiles between two groups, except for platelets, which were significantly elevated in the TPOm treated mice by day 14. **(D–F)** Incidentally, total CD45+ lineage- cKit+ Sca1+ (LSK) cells, CD45+ lineage-Sca1-cKit+CD41+ Megakaryocyte Progenitor (MKP) and CD45+ lineage-Sca1-cKit+ (LK) cells was significantly higher in TPOm treated mice by day 14 or day 30 post BMT, respectively. Data are expressed as mean ± SEM. Significance levels: ns = not significant, *p < 0.05, **p < 0.01, ***p < 0.001, ****p < 0.0001.

First, we evaluated the effect of TPOm on donor chimerism in a congenic BMT mice model. This evaluation was performed in transplanted mice groups treated with PBS and 300µg/Kg TPOm. By day 30 post-BMT, both PBS and TPOm treated mice showed ≥80% donor (CD45.1) cells in BM, indicating successful engraftment (**Figure 1A**). However, total number of BM cells (**Supplementary Figure S1**) and total donor cells was higher in TPOm treated mice (**Figure 1B**). CBC analysis of peripheral blood showed no major difference between PBS and TPOm treated mice except that TPOm treatment significantly increased platelets by day 14 post BMT (**Figure 1C**). Further, TPOm treated mice showed a significant increase in total donor CD45+ lineage-Sca1-cKit+ (LK) cells, CD45+ lineage- cKit+ Sca1+ (LSK) cells, CD45+ lineage-Sca1-cKit+CD41+ Megakaryocyte Progenitor (MKP) cells over PBS treated mice (**Figures 1D–F**). The increase in MKP supported the platelet increase in peripheral blood post TPOm treatment. The data clearly indicates that TPOm is safe to be used in BMT setting and does not negatively affect donor engraftment. Additionally, TPOm could benefit BMT by expanding hematopoietic stem and progenitor cells, thereby enhancing bone marrow recovery and function.

### JNJ-26366821 dose-dependently accelerates BM vascular regeneration

**Figure 2:**
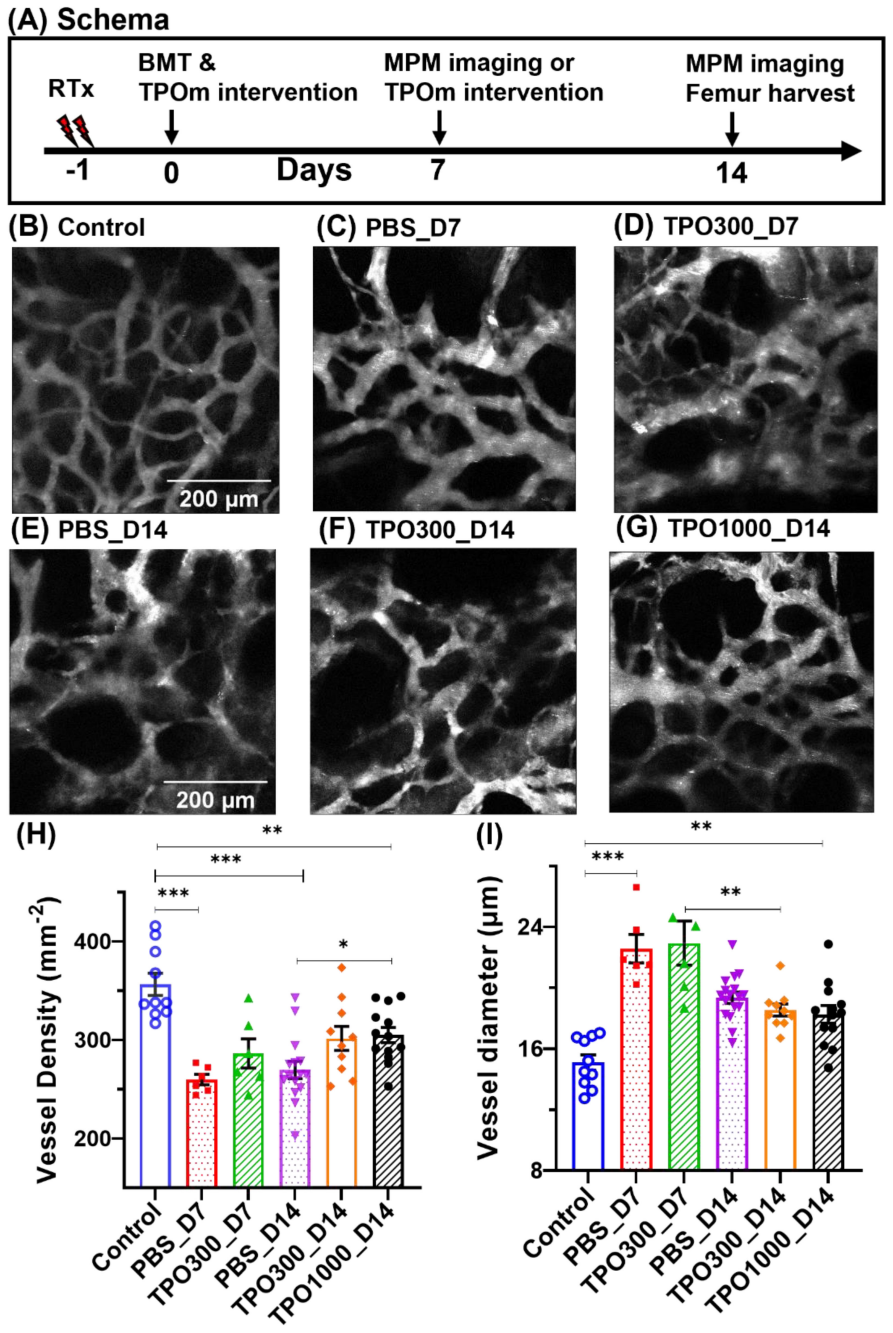
BM vessels in the frontal calvarium bone, showing changes in vascular characteristics and their recovery during the healing process. **(A)** Schema of the experimental design. **(B)** The BM vasculature of a control mouse as seen in intravital microscopy, where the structural basis of a complete vascular network can be observed. **(C–G)** Images of transplant mice, given 11 Gy x-ray irradiation followed by BMT and TPOm administration. Irradiation caused several vascular anomalies, including swelling or vasculopathy; a complex network of tortuous vessels of various sizes was observed. **(C)** BM vascular images of transplant mice at day 7 of post-BMT, which are injected with PBS (0 µg/kg TPOm). **(D)** The same group of transplant mice was injected with TPOm (300 µg/kg body weight) within an hour of BMT. **(E–G)** Representative images of transplant mice at day 14, injected with PBS, 300 µg/Kg TPOm and 1000 µg/Kg TPOm, respectively. Day 14 groups received a second dose of TPOm or PBS 7 days after BMT. **(H)** Average vessel density, MVD per mm^2^ of the imaging region, with group sizes of n = 10, 6, 6, 16, 10, and 12 mice, respectively, per group. **(I)** The average vessel diameter. Average diameter values came closer to control mice on day 14, compared to day 7 as a manifestation of vascular recovery along with the time elapsed since injury. Similarly, at day 14 post-BMT, significant differences in MVD can be seen between PBS and 1000 g/Kg TPOm treated mice, demonstrating that TPOm treatment improves vascular recovery. Two-way ANOVA with Tukey’s multiple comparisons and BH correction was used. Data are shown as mean ± SEM, with significance set at p < 0.05. Significance levels: *p < 0.05, **p < 0.01, ***p < 0.001.

After validating TPOm was not deleterious for BMT, we next evaluated dose dependent effect of TPOm on BM vasculature post BMT. The BM vascular structure and functional recovery was assessed on day 7 and day 14 post BMT in mice treated with PBS and different doses of TPOm (300μg and 1000μg/kg). Microvascular density (MVD) showing mean blood vessel number per mm^-2^ imaging area of MPM, and average BM vessel diameter were first evaluated to assess vascular regeneration. **Figure 2A** shows the schema of the experimental design. The vascular networks of control (non-irradiated healthy control group) mice are more smoother (**Figure 2B**), whereas those of mice that received radiation conditioning followed by BMT are dilated and tortuous (**Figures 2C–G**). Among the transplant group, vessels dilation is less on day 14 (**Figures 2E–G**) compared to day 7 (**Figures 2C, D**). On day 14, the MVD in the irradiated animals showed a trend towards recovery in vascular density and diameter consistent with the baseline in the non-irradiated control group with the recovery being less in the PBS-treated (**Figure 2E**) compared to the TPOm-treated (**Figures 2F, G**). The average MVD (**Figure 2H**) and vascular diameters (**Figure 2I**) change as a function of irradiation time, and TPOm dose. Interestingly, the number of blood vessels in the imaging area of 1000 µg/Kg TPOm administered on day 14 groups (TPO1000_D14) significantly (P = 0.027) shifted from their corresponding irradiated control types (PBS_D14) towards non-irradiated healthy controls (Control).

### JNJ-26366821 dose-dependently accelerates BM vascular physiological restoration

**Figure 3:**
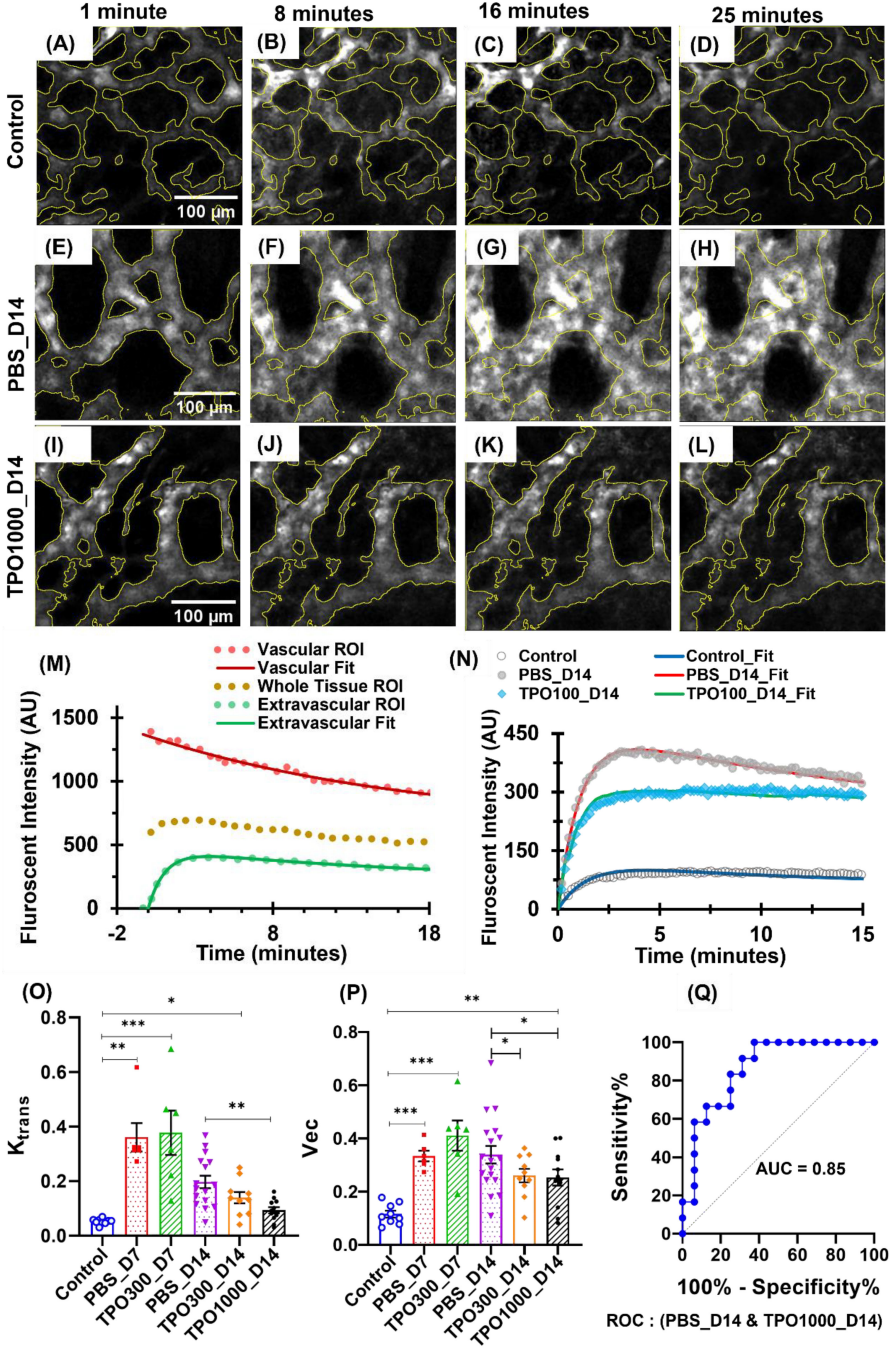
Temporal variation of 150 KDa TRITC-dextran fluorescence at BM vessels of the frontal calvarium bone after different intervals of intravenous injection. **(A–L)** Representative time-lapse images showing the physiological restoration of BM vasculature in transplant mice with and without TPOm intervention as reflected in contrast extravasation rate. **(A–D)** Control mice images, within 1 minute, after 8 minutes, after 16 minutes, and after 25 minutes of contrast injection. **(E–H)** PBS intervened transplant mice images, within 1 minute, after 8 minutes, after 16 minutes, and after 25 minutes of contrast injection. **(I–L)** 1000 µg/kg TPOm intervened transplant mice images, within 1 minute, after 8 minutes, after 16 minutes, and after 25 minutes of contrast injection. **(M)** Tissue kinematics curves showing fluorescent intensity values of vascular tissue, extravascular tissue, and the whole tissue ROIs of time-lapse images. Fitted curves are used to evaluate the kinematic parameters. **(N)** Representatives of time-lapsed dextran fluorescent intensity from the extravascular tissue compartment regions for the control, PBS treated and TPOm treated irradiated mice. **(O)** Transfer rate (K_trans_) of contrast molecules between blood plasma and extracellular extravascular tissue. The group sizes are n = 9, 6, 6, 16, 10, and 12 mice, respectively, per group. 1000 µg/kg TPOm treatment has significantly improved their vascular physiology compared to mice without TPOm treatment. **(P)** Assessment of the fractional volume of contrast in EES (*v_ec_*). Two-way ANOVA with Tukey’s multiple comparisons and BH correction was used. Data are shown as mean ± SEM, with significance set at p < 0.05. Significance levels: *p < 0.05, **p < 0.01, ***p < 0.001. **(Q)** ROC curve and corresponding AUC value while comparing K_trans_ values between PBS_D14 and TPO1000_D14.

To measure BMT-induced changes in BM vascular morphology and functions, we have analyzed the time-lapse images using 150 kDa TRITC-dextran contrast influx from plasma into extracellular and extravascular tissue. Representative time-lapse images of vascular networks showing the temporal variation of contrast after 1 minute, 8 minutes, 16 minutes, and 25 minutes of the intravenous contrast agent injection are shown in (**Figures 3A–L**). TRITC-dextran extravasation with time is higher in vasculature of irradiated PBS treated mice (**Figures 3E–H**) compared to control mice (**Figures 3A–D**). However, contrast extravasation rate in TPOm intervened mice (**Figures 3I–L**) are relatively closer towards control mice (**Figures 3A–D**).

Physiological alteration of the BM vasculature was evaluated by characterizing the time-intensity curves of the sequential contrast distribution (time-lapse) images using Toft’s two compartmental modeling approaches as discussed (30, 31). Herein, the fluorescent intensity curves showing kinematics of contrast in tissue (vascular, extravascular, and whole tissue) regions are shown in (**Figure 3M**). Irradiation causes vascular damage, resulting in vascular dilation, increased extravasation rates and extravascular tissue uptake of 150 kDa TRITC-dextran (**Figure 3N**). (**Figure 3O**) shows transfer rate (K_trans_) values are increasing significantly after BMT, but they recover with time. The results of our study also show the restoration rate of vascular physiology is significantly improved in 1000 µg/kg TPOm-treated mice on day 14, compared to their corresponding controls treated with PBS. A similar trend can be seen in the fractional volume of EES (*v_ec_*) assessment, with a significant difference (P ~ 0.035) in PBS_D14 and TPO1000_D14 (**Figure 3P**).

Additionally, the discriminating potential K_trans_ values between PBS_D14 and TPO1000_D14 were further analyzed with statistical measures such as sensitivity, specificity, and the receiver operating characteristic (ROC) curve, as shown in (**Figure 3Q**). The discriminatory signatures reveal a promising application of TPOm therapy to improve vascular health in transplant mice with a sensitivity of 84%, specificity of 71%, and area under the curve (AUC) 85% when comparing K_trans_ values.

### JNJ-26366821 treatment in combination with BMT following irradiation improves vasculature of femur BM

**Figure 4:**
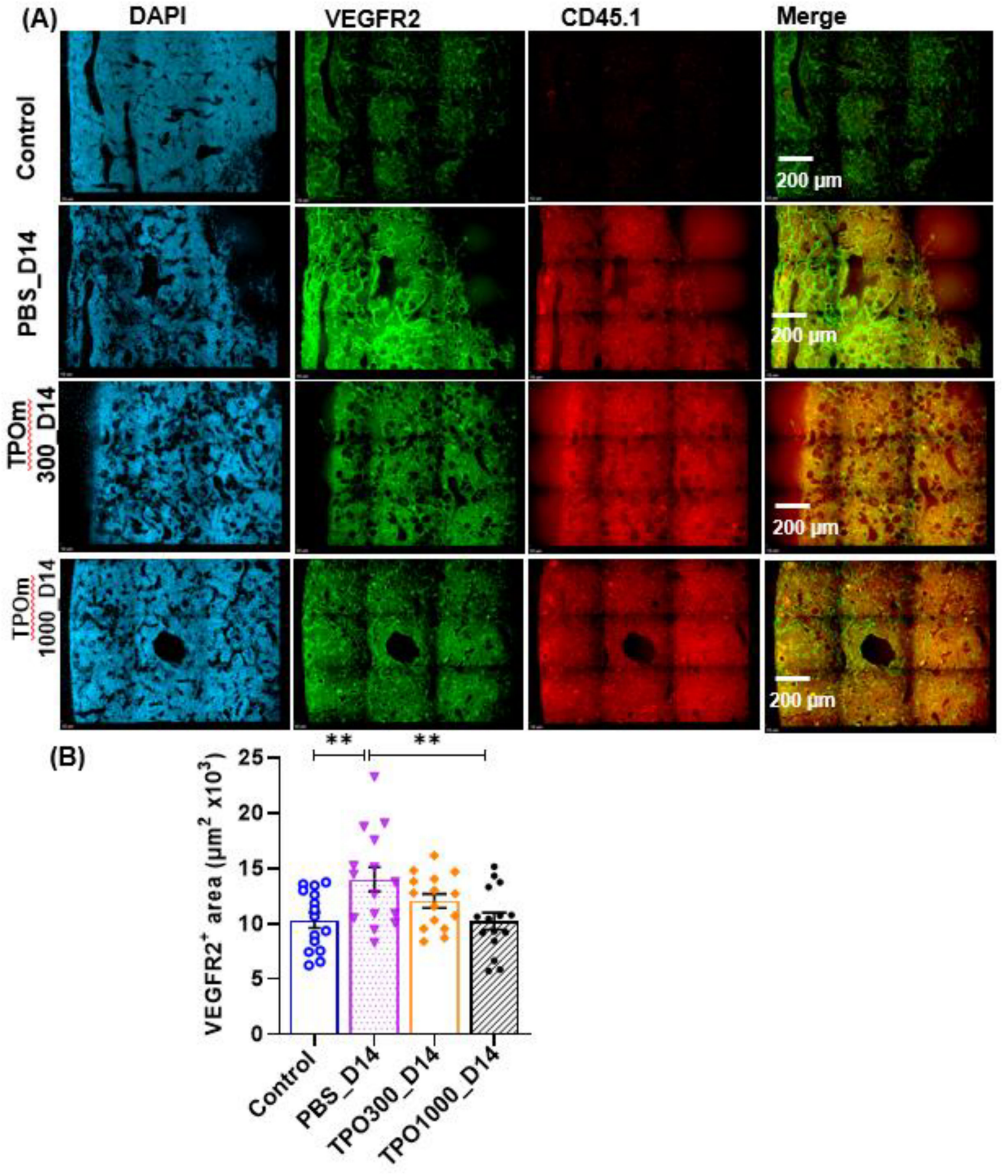
TPOm treatment with BMT after lethal irradiation ameliorates vascular dilation in the bone marrow. **(A)** Representative immunofluorescent images of femurs stained with DAPI (blue) and VEGFR2 (green) on day 14 after lethal irradiation. **(B)** Quantification of surface area of VEGFR2^+^ vessels of in the bone marrow (n=3/group). Data are presented as mean ± SEM, with ** indicating p < 0.01, a statistically significant difference.

Image-based histological methods are widely used for the analysis of vasculature and its microenvironment within the tissue specimens (34). Sinusoids in the diaphysis of the femoral BM were analyzed by whole-mount confocal microscopy. (**Figure 4A**) are representative images of the BM sinusoids of the BM niche stained with DAPI (blue), VEGFR2 for all vessels(green), and donor transplant cells with CD45.1 (red). Despite BMT, PBS-treated animals visually had more dilated vessels than TPOm-treated animals. quantified by assessing the complete VEGFR2^+^ area per field (**Figure 4B**). Further, the VEGFR2^+^ area was significantly lower (P-value < 0.01) in the 1000 µg/kg TPOm treated group compared to PBS-treated (**Figure 4B**).

## Discussion

The findings of our study show that JNJ-26366821 (TPOm) intervention clearly showed that TPOm is safe to be used in a BMT setting and it would improve donor hematopoietic stem and progenitor cell (HSPCs) expansions ensuring good engraftment. Further, TPOm has a dose- and time-dependent effect on improving the morphological and biophysical performance of the BM vasculature in mice treated with a myeloablative radiation conditioning regimen for BMT. Herein, we examined both morphological (average diameter and MVD) and biophysical (kinematics of contrast flow: K_trans_ and *v_ec_*) parameters of the BM vasculature in live mice at microscopic resolution using MPM to measure the impact of the TPOm on BM vascular recovery. JNJ-26366821-treated mice showed significant improvements in MVD, contrast extravasation, K_trans_, and *v_ec_*, compared to those treated with PBS. Whole-mount confocal microscopy images of the femur BM further confirmed enhanced vascular regeneration in the JNJ-26366821-treated group. These findings suggest that JNJ-26366821 promotes BM vascular recovery in mice undergoing myeloablative radiation conditioning for BMT, offering potential therapeutic benefits in the field of HCT.

TPOm administration increases hematopoietic stem and progenitors’ cells (HSPCs) in a control B6 mice (Data not shown). In this study, we showed that TPOm administration also increases HSPCs (LSK, LK and MKP) in a BMT mouse model. This increase in donor HSPCs also results in increased BM cellularity in TPOm treated mice suggesting augmented hematopoietic recovery post BMT particularly platelets. Delayed platelet recovery is an important complication in allogeneic Hematopoietic stem cell transplant (HCT) patients. Therefore, TPOm could be a safe treatment option to enhance hematopoietic recovery in allogenic HCT patients. Although we observe increased short-term engraftment, further studies need to be conducted to understand the effect of TPOm on long-term engraftment.

In addition, the ability of TPOm intervention to accelerate vascular recovery after BMT may have wide applications in improving hematological disorders, including malignancies requiring stem cell transplantation. In TMLI dose escalation (20 Gy) treatment compared to standard myeloablative TBI (13.2 Gy)-based regimens shows a significant improvement in 2-year overall survival (41% vs. 15%) in young adult (18-55 years old) with active disease (11, 12). Additionally, TMLI (16 Gy) conditioning to older populations (18-55 years old) (NCT03494569) has demonstrated promising 2-year overall survival rates (64%). In a preclinical TMLI-BMT model, Lim et al. also observed the feasibility of dose escalation (16 Gy) for old mice (35). However, the impact of increased radiation dose on post-BMT BM vascular environment is unknown. If future correlative investigations suggest adverse effects on BM vascular recovery, despite these advanced TMLI-based conditionings, JNJ-26366821 intervention may become an option for normalizing the bone marrow vascular environment and facilitating rapid donor stem cell engraftment post-HCT (36).

Another promising area of study that could benefit from JNJ-26366821 intervention is sickle cell disease (SCD) (37). SCD presents challenges due to microvascular occlusions by sickle red blood cells (RBCs), leading to complications like vaso-occlusion, oxidative stress, ischemia-reperfusion injury, and inflammation (38). Allogeneic HCT is the current curative treatment for SCD (39). Reduced-intensity conditioning (RIC) (40) minimizes organ toxicity, but it often lowers the levels of donor chimerism leading to a high risk of ongoing SCD-associated events and graft failure (41, 42). In preclinical study, Srideshikan et al. demonstrated that BM targeted radiation dose escalation (8 Gy) in mice using TMLI improved chimerism and long-term engraftment by rescuing from SCD (43). A clinical phase I trial (NCT05384756) was also initiated to assess the safety and efficacy of using TMLI (6 Gy) and alemtuzumab as a conditioning regimen for HCT in SCD patients with matched donors. However, there is also a limit to the increase of radiation to BM, as it may adversely impact the host BM stroma supporting donor engraftment. Thus, combining TMI with TPOm could be a potential strategy to augment regeneration of vascular system and improve chimerism.

There are several limitations to the current study. Firstly, the assessment of the BM vasculature using MPM was limited to the cranial bone marrow, which may not fully represent changes in the entire skeletal system due to potential spatial heterogeneity (44). To address this limitation, we further validated the response to the JNJ-26366821 intervention using the histology of femoral bone marrow. Both the femur and skull bone marrow regions show similar concurrent improvements in BM vasculature with JNJ-26366821 intervention. Another limitation is the lack of longitudinal monitoring of the disease burden and drug response in the same mice over time for a better assessment of biological complexity. The implant-tissue interface (used for the cranial window) degrades with mucosal/granulation-tissue formation over time, so the temporal variation of disease response may not be monitored in the same groups of mice prepared in the same environments. Additionally, our current study focuses on congenic transplants, limiting the scope of immune cell induced toxicity, a major complication observed in allogenic HCT. Future research could explore allogeneic BMT to understand the role of augmented vascular recovery on engraftment and Graft vs host response. The role of other contributing cells in BM, such as stromal niches, hematopoietic stem and progenitor cells (HSPCs) can also be explored, as our collaboration has done earlier in non-BMT settings of an acute radiation syndrome mouse model (45).

In summary, the findings of this study demonstrate that JNJ-26366821 administration enhances BM vasculature and microenvironment recovery in mice that have undergone myeloablative radiation conditioning for BMT. Moreover, no acute adverse effects were observed in JNJ-26366821-treated mice post-BMT, providing additional preclinical evidence for future clinical testing. These promising results support further evaluation of the use of JNJ-26366821 in a clinical study of BMT patients.

## Data Availability

The raw data supporting the conclusions of this article will be made available by the authors, without undue reservation.
