# Supplementary material for: Thrombopoietin mimetic therapy alleviates radiation-induced bone marrow vascular injury in a bone marrow transplant mouse model
Source: Front Oncol. 2024 Oct 10;14:1414488. doi: 10.3389/fonc.2024.1414488 (PMC11499237; doi:10.3389/fonc.2024.1414488)
Supplement: Supplementary file 1 [file DataSheet1.docx]

**Thrombopoietin mimetic therapy alleviates radiation-induced vascular injury in a bone marrow transplant mouse model**

Hemendra Ghimire, Srideshikan Sargur Madabushi, Justin Vercellino, Jamison Brooks, Darren Zuro, Ji Eun Lim, Paresh Vishwasrao, Amr Mohamed Hamed Abdelhamid, Guy Strome, Gary Eichenbaum, Monzr Al Malki, Chandan Guha, and Susanta K. Hui

**Supplementary Introduction**

**JNJ-26366821:**

JNJ-26366821 (TPOm), a pegylated synthetic peptide distinct from endogenous thrombopoietin, is being developed to address platelet recovery failure after HSC transplantation[1]. It has shown promising efficacy in improving survival outcomes and hematopoietic recovery in mice exposed to radiation[2]. The intervention enhances recovery of indicators such as BM colony forming units, megakaryocytes, and FMS-like tyrosine kinase 3 ligand (FLT3-L). TPOm treatment in ear veins of radiation exposed mice has shown it’s potential in reducing associated vascular leakage and lowering inflammation, as observed through a decrease in leukocyte-endothelial cell interactions[3]. Additionally, in a pilot study using a rat model of prostate radiation therapy, TPOm treatment exhibits significant vascular protective effects, preventing changes in the penile artery cross-sectional area induced by radiation therapy[4]. These findings suggest the potential of JNJ-26366821 to reduce toxicities from radiotherapy and safeguard crucial microvascular structures for tissue function. Studies also indicate that JNJ-26366821 treatment increases megakaryopoiesis without affecting malignant myeloid proliferation in myelodysplastic syndrome (MDS) and acute myeloid leukemia (AML)[1]. This selective effect is particularly valuable in BMT, where achieving a balance between hematopoietic recovery and controlling malignancy is crucial[5].

**Supplementary Result**

**Total number of bone marrow cells:**

The total number of bone marrow cells was evaluated in TPOm and PBS treated mice on days 7, 14, and 30 post-BMT. As shown in Figure S1, the TPOm treated mice exhibited a significant increase in the total number of bone marrow cells compared to the PBS treated mice on day 30.


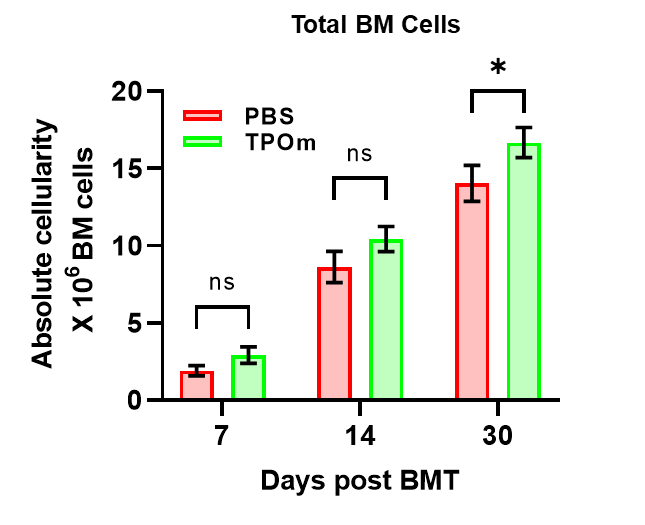


**Figure S1**: Total number of Bone marrow cells in transplanted mice at D7, 14 and D30 post-BMT. There is a significant difference in bone marrow cells in TPOm and PBS treated mice on day 30. Data are expressed as mean ± SEM.

**Supplementary 1: Key resource table**

| **Reagent type (species) or resource** | **Designation** | **Source or reference** | **Identifiers** | **Additional information** |
| --- | --- | --- | --- | --- |
| Chemical compound | Sucrose | Sigma-Aldrich | Cat#: S0389 | Final conc: 30% w/v |
| Chemical compound | O.C.T Compound | Fisher | Cat#: 23-730-571 |  |
| Chemical compound | DMSO | Corning | Cat#: 25-950-CQC | Final conc: 10% v/v |
| Chemical compound | Triton X-100 | Sigma-Aldrich | Cat#: T8787 | Final conc: 0.5% v/v |
| Chemical compound | Hoechst 33342, trihydrochloride. trihydrate | Life Technologies | Cat#: H3570 | IF (1:2000) |
| Other | Horse Serum | Gibco | Cat#: 16050130 | Final conc: 5% v/v |
| Antibody | Anti-mouse VEGFR2 (Goat polyclonal) | R&D Systems | Cat#: AF644 | IF (1:100) |
| Antibody | Alexa Fluor 594 anti-mouse CD45.1 (mouse monoclonal) A20 | Biolegend | Cat#: 110750 | IF (1:100) |
| Antibody | Alexa Fluor 488 AffiniPure F(ab')₂ Fragment Donkey Anti-Goat IgG (H+L) | Jackson ImmunoResearch | Cat#: 705-546-147 | IF (1:500) |
| Software | Volocity | Quorum Technologies |  | v6.5.1 |
| Software | Prism | GraphPad |  | v9.4.1 |
| Software | Bitplane Imaris | Oxford Instruments |  | v9.6.0 |

**Table S1.** Key resource table for the whole-mount immunofluorescence imaging and analysis.

**Supplementary References**

1. Adrianzen-Herrera, D., et al., *The thrombopoietin mimetic JNJ-26366821 increases megakaryopoiesis without affecting malignant myeloid proliferation.* Leukemia & Lymphoma, 2020. **61**(10): p. 2453-2465.

2. Kumar, V.P., et al., *Mitigation of total body irradiation-induced mortality and hematopoietic injury of mice by a thrombopoietin mimetic (JNJ-26366821).* 2022. **12**(1): p. 3485.

3. Ashcraft, K.A., et al., *Application of a novel murine ear vein model to evaluate the effects of a vascular radioprotectant on radiation-induced vascular permeability and leukocyte adhesion.* 2018. **190**(1): p. 12-21.

4. Ashcraft, K.A., et al., *Clarifying the relative impacts of vascular and nerve injury that culminate in erectile dysfunction in a pilot study using a rat model of prostate irradiation and a thrombopoietin mimetic.* 2019. **103**(5): p. 1212-1220.

5. Pinho, S. and P.S.J.N.r.M.c.b. Frenette, *Haematopoietic stem cell activity and interactions with the niche.* 2019. **20**(5): p. 303-320.
